# Second-Trimester Uterine Rupture in a Multiple-Scar Uterus Complicated by Placenta Accreta Spectrum: A Case Report and Literature Review

**DOI:** 10.7759/cureus.108892

**Published:** 2026-05-15

**Authors:** Stylianos Sergios Chatziioannou, Varvara Papasideri, Pantelis Palaiologos

**Affiliations:** 1 School of Medicine, European University of Cyprus, School of Medicine, Nicosia, CYP; 2 Healthcare Center, The JBI (Joanna Briggs Institute) University of West Attica Evidence-Based Healthcare Center, Athens, GRC; 3 First Department of Obstetrics and Gynecology, Elena Venizelou General Maternity Hospital, Athens, GRC; 4 School of Psychology, European University of Cyprus, Nicosia, CYP; 5 Department of Obstetrics and Gynaecology, Larnaca General Hospital, Larnaca, CYP

**Keywords:** emergency laparotomy, extrauterine intact amniotic sac, placenta accreta spectrum, second-trimester pregnancy, uterine rupture

## Abstract

Uterine rupture is a rare but life-threatening obstetric emergency, most commonly occurring in women with a scarred uterus. Placenta accreta spectrum (PAS) increases the risk of uterine wall disruption and severe hemorrhage, while spontaneous rupture before labor, particularly in the second trimester, remains uncommon. We report a 28-week pregnant woman with two prior cesarean sections who presented after a fall with abdominal pain and vaginal bleeding. Evaluation revealed intrauterine fetal demise and findings consistent with uterine rupture. Emergency laparotomy confirmed a complete rupture at the previous cesarean scar, with an intact extrauterine amniotic sac. Subtotal hysterectomy was required due to hemorrhage, and histopathology confirmed PAS. Early recognition and multidisciplinary management were essential for maternal stabilization.

## Introduction

Uterine rupture is a well-recognized obstetric emergency characterized by complete disruption of the uterine wall, resulting in communication between the uterine cavity and the peritoneal space [1,2]. This event is associated with significant maternal and fetal morbidity and mortality, particularly when it occurs outside of labor. The risk of uterine rupture increases substantially in women with a history of cesarean delivery, with the likelihood rising with the number of prior uterine surgeries [1,3].

Placenta accreta spectrum (PAS) describes a range of disorders in which the placenta abnormally adheres to or invades the myometrium [4,5]. The incidence of PAS has risen in parallel with increasing cesarean rates globally. PAS is a known contributor to uterine wall weakening and may predispose to rupture, especially when implantation overlies a previous cesarean scar. Most commonly, PAS is associated with severe hemorrhage at the time of attempted placental separation during labor or delivery [6,7]. However, it may also contribute to uterine dehiscence or rupture before the onset of labor [6,8].

Second-trimester rupture remote from term is rare [1,3]. It has been described in isolated cases, often in the setting of scarred uteri with abnormal placentation [8,9]. Clinical features include sudden-onset abdominal pain, vaginal bleeding, loss of fetal heart activity, and signs of intra-abdominal bleeding [4]. Diagnosis is typically clinical, supported by imaging, and confirmed at laparotomy.

An exceptional and rarely reported phenomenon is the extrusion of an intact amniotic sac containing a demised fetus into the peritoneal cavity following complete uterine rupture [8,9]. In such cases, despite full-thickness myometrial disruption, the membranes remain preserved, and the fetus is contained within the sac. When PAS is present, invasive placental tissue may lead to inflammatory adhesion formation between the extruded sac and adjacent viscera, increasing surgical complexity [6,7].

Here, we describe a case of uterine rupture at 28 weeks’ gestation in a woman with two prior cesarean sections and no antenatal care. The rupture resulted in the presence of an intact extrauterine amniotic sac adherent to intra-abdominal structures and was managed with emergency laparotomy, subtotal hysterectomy, and left adnexal resection. We also review the literature on second-trimester uterine rupture and discuss the implications of PAS in this rare clinical scenario.

## Case presentation

In 2025, a pregnant woman at approximately 28 weeks’ gestation presented to the emergency department of the General Hospital of Larnaca with abdominal pain and vaginal bleeding. She reported that her last menstrual period was on January 15, 2025. The patient stated that she had slipped in the bathroom shortly before the onset of symptoms.

On initial evaluation, her vital signs were stable. Blood pressure was 130/90 mmHg, heart rate was 80 beats per minute, temperature was 36 °C, and oxygen saturation was 100% on a fraction of inspired oxygen (FiO₂) of 24%. She complained of persistent abdominal pain accompanied by vaginal bleeding. Laboratory evaluation on admission revealed a hematocrit of 27.8%, hemoglobin of 9.0 g/dL, white blood cell count of 4.7 × 10³/μL, and platelet count of 389 × 10³/μL.

Her obstetric history was significant for two previous cesarean sections. She had not attended any antenatal visits and had not been evaluated by a gynecologist during the current pregnancy. Ultrasound examination demonstrated a 28-week intrauterine pregnancy without fetal cardiac activity. The initial ultrasound examination confirmed intrauterine fetal demise without definitive sonographic evidence of uterine rupture or significant hemoperitoneum. Given the confirmed fetal demise and the patient’s initially stable hemodynamic condition, conservative inpatient management with induction was initiated using two sublingual tablets of misoprostol (Cytotec), along with antibiotic therapy and analgesics. The patient was admitted for close monitoring and further management.

During the night following admission, the patient continued to report persistent abdominal pain and developed recurrent brownish vaginal bleeding. Due to the persistence of symptoms, a repeat ultrasonographic evaluation was performed during the early morning hours by the director of the clinic. The repeat ultrasound demonstrated findings highly suspicious for uterine rupture with extrauterine displacement of the gestational sac. Immediate preparation for surgical intervention was therefore initiated, and three units of packed red blood cells were requested from the blood bank.

**Figure 1:**
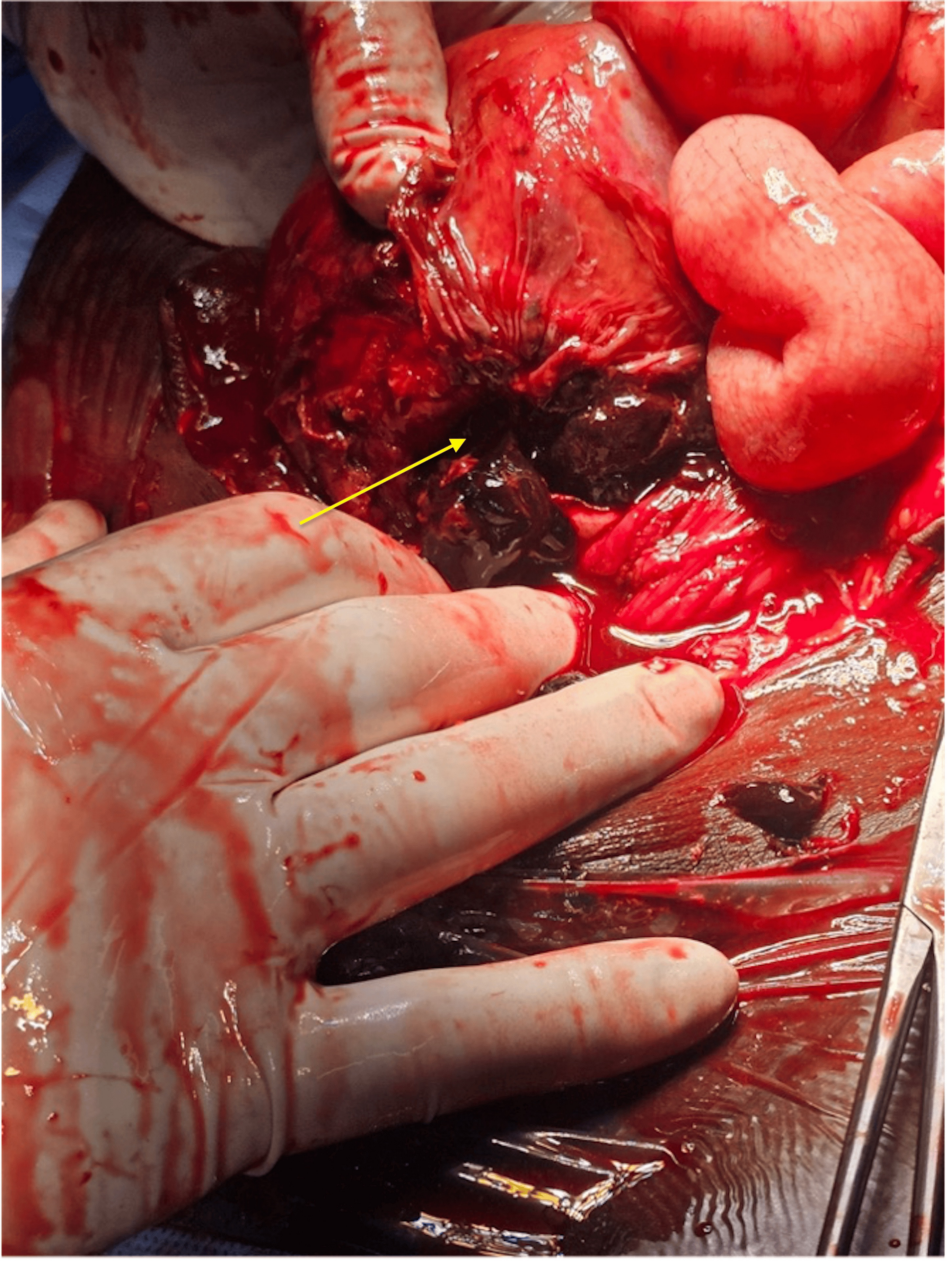
Rupture of the uterine cavity in the previous C-section (yellow arrow)

**Figure 2:**
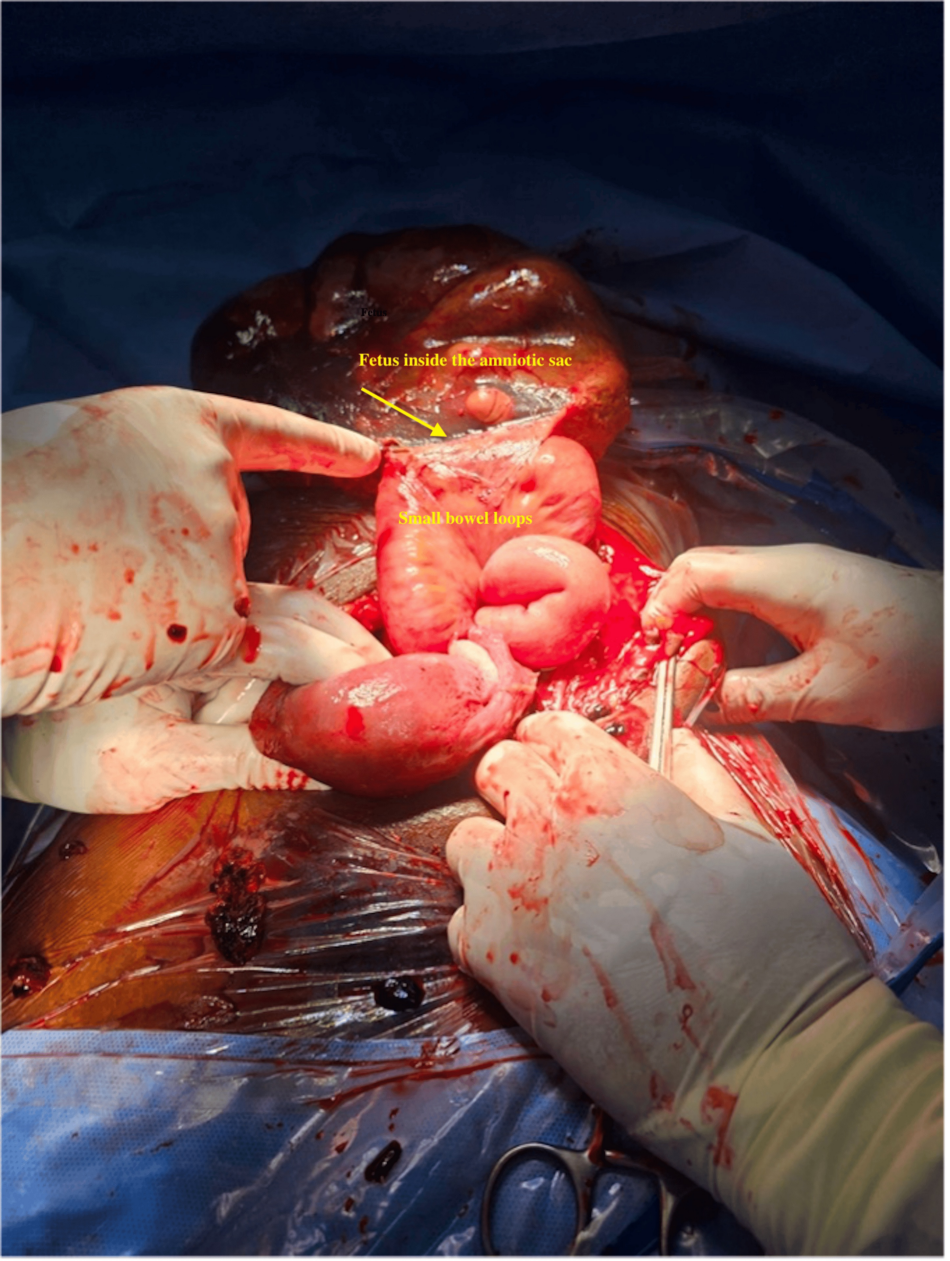
At the entrance of the peritoneal cavity, the amniotic sac is adherent to the small bowel loops.

Emergency laparotomy was performed without delay. Upon entry into the peritoneal cavity, complete rupture of the uterus was identified at the site of the previous cesarean section scar (Figures 1, 2). The intact amniotic sac containing the demised fetus was located entirely outside the uterine cavity within the abdominal cavity. The sac was densely adherent to loops of small bowel, the omentum, and the anterior parietal peritoneum. Hemoperitoneum was present, and active bleeding was noted from the ruptured scar and placental bed.

**Figure 3:**
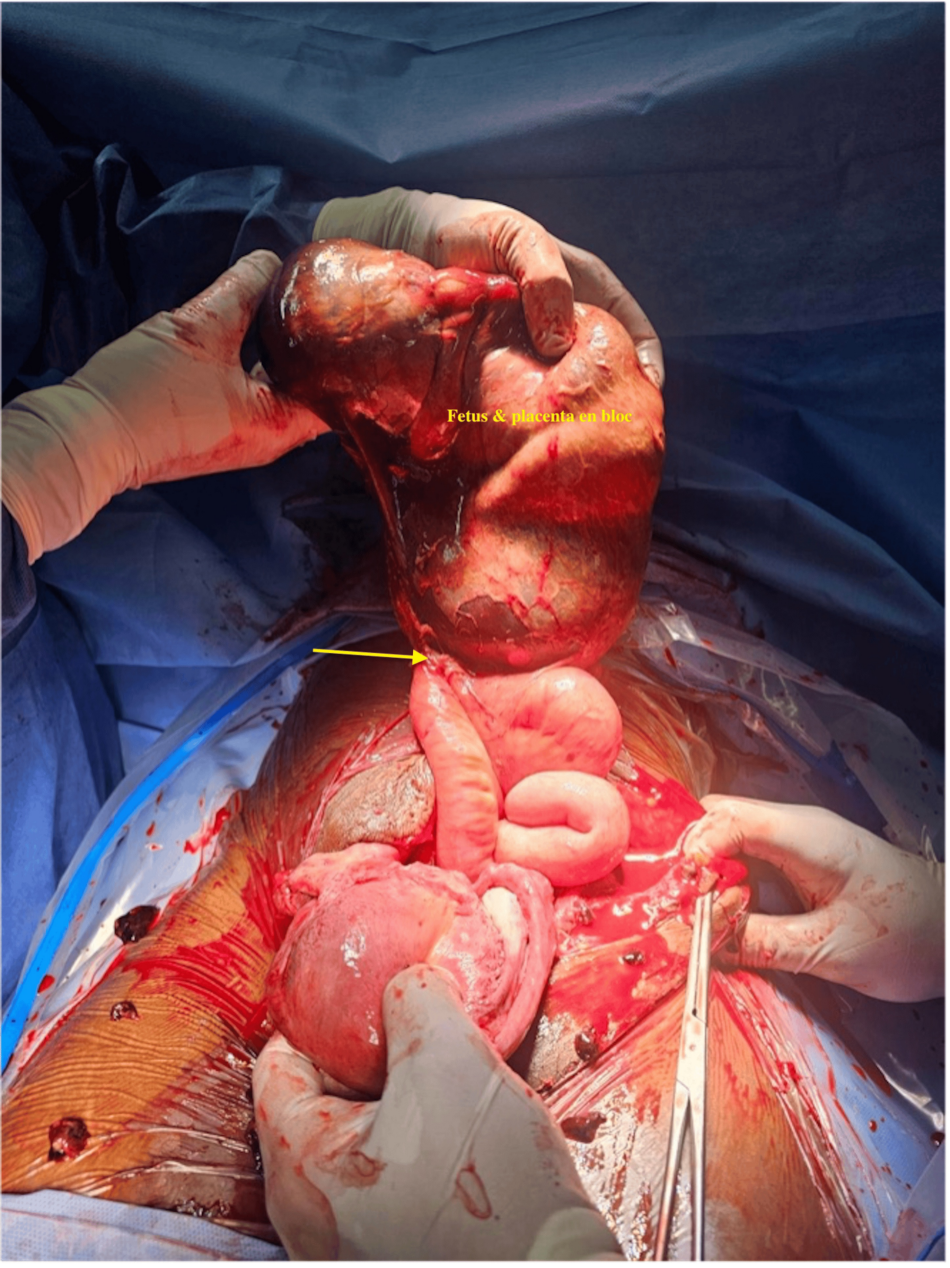
Fetus and placenta are en-block adherent to the small bowel loops (yellow arrow) outside the uterine cavity.

Due to the intimate involvement of small bowel loops with the amniotic sac, a general surgeon was called intraoperatively to carefully separate the adhesions and prevent enteric injury (Figure 3). After opening the intact amniotic sac and delivering the fetus, persistent hemorrhage and extensive destruction of the lower uterine segment necessitated definitive surgical management. A subtotal hysterectomy was performed. Resection of the left adnexa was also required because of ongoing bleeding in the surgical field. A portion of the uterus and the left adnexa were sent for histopathological examination. A peritoneal drain was placed and remained in situ for three days.

**Figure 4:**
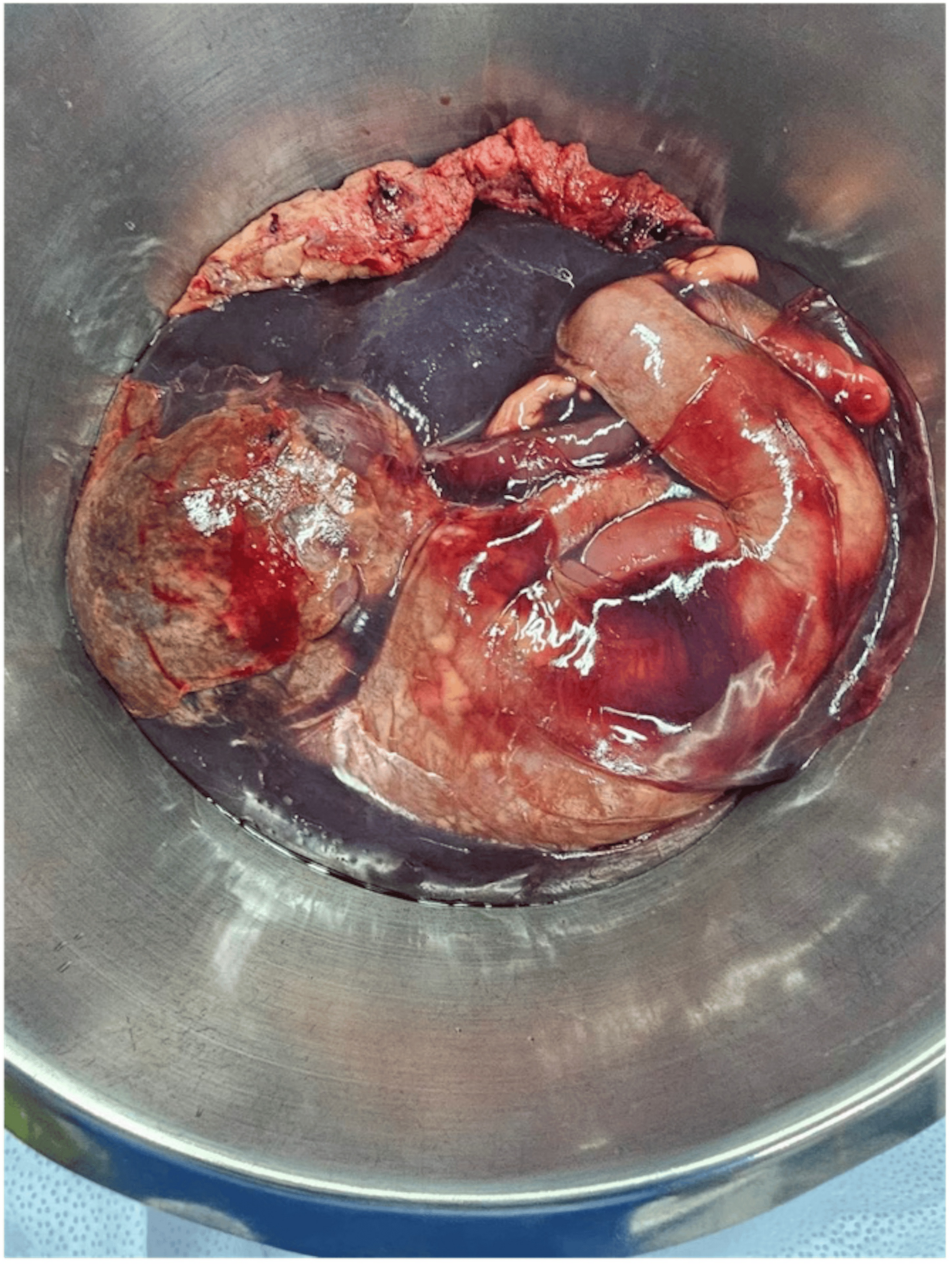
Gross surgical specimen demonstrating the fetus and placenta en bloc following removal during emergency laparotomy. Histopathological evaluation of the surgical specimen subsequently confirmed placenta accreta spectrum involving the previous cesarean scar region. Histopathological images were not available for inclusion in the present report.

During the operation, two of the three requested units of packed red blood cells were transfused. Histopathological examination demonstrated abnormal chorionic villous adherence directly to the myometrium with marked thinning and disruption of the uterine wall in the region of the previous cesarean scar. Decidual deficiency was identified within the scar area, consistent with placenta accreta spectrum pathology. Extensive myometrial destruction and hemorrhagic changes were present adjacent to the rupture site (Figure 4). No alternative uterine pathology was identified. The specimens were sent for histopathologic evaluation.

Postoperative course

The surgical procedure was successful. The patient remained hemodynamically stable postoperatively and did not develop complications related to bowel injury. She remained hospitalized for six days. At discharge, she was instructed to administer prophylactic anticoagulant therapy for seven days and to return for removal of abdominal wound clips seven days after hospital discharge. She was discharged in stable condition.

## Discussion

Compared with previously reported cases, the present case demonstrates several clinically important features that increase both diagnostic complexity and surgical risk. Although second-trimester uterine rupture has been described in women with previous cesarean sections, the coexistence of histopathologically confirmed placenta accreta spectrum (PAS), complete extrusion of an intact extrauterine amniotic sac, and dense adhesions involving bowel structures remains exceptionally uncommon [10,11]. In contrast to many previously published cases in which rupture was identified rapidly after hemodynamic collapse, our patient initially presented in a relatively stable condition despite significant intra-abdominal pathology. This emphasizes that uterine rupture may initially manifest with subtle or nonspecific findings, particularly in patients with abnormal placentation and prior uterine surgery.

Epidemiology of uterine rupture in scarred uteri

Uterine rupture is a rare but severe obstetric complication, most commonly occurring in women with a prior cesarean section. The likelihood of rupture rises proportionally with the number of prior cesarean sections due to progressive thinning and structural weakening of the lower uterine segment.

Second-trimester or early third-trimester uterine rupture is distinctly uncommon, as most cases occur during labor. When rupture occurs before labor, it is frequently associated with abnormal placentation, particularly PAS. In such cases, invasive placental implantation may progressively compromise myometrial integrity, predisposing the uterus to spontaneous rupture [5].

In the present case, the patient had two previous cesarean sections and no antenatal surveillance. Although the patient reported a preceding fall before symptom onset, the intraoperative and histopathological findings suggest that placenta accreta spectrum and previous cesarean scar weakening were likely the principal underlying predisposing factors for rupture. The traumatic event may have acted as a precipitating trigger in an already structurally compromised uterus rather than representing the sole etiologic mechanism. Similar observations have been described in previous reports where minimal trauma or increased intra-abdominal pressure preceded rupture in patients with abnormal placentation and scarred uteri.

Placenta accreta spectrum and risk of early uterine rupture

PAS comprises abnormal placental attachment disorders characterized by excessive trophoblastic invasion into the myometrium [4,5]. The global incidence of PAS has increased in parallel with rising cesarean delivery rates and is currently estimated to occur in approximately 1 in 300-500 pregnancies in high-resource settings [5,10].

When PAS overlies a previous cesarean scar, the myometrial layer may become markedly thinned or completely absent. This structural compromise may predispose to uterine dehiscence or complete rupture, even before the onset of labor. Although PAS is most commonly associated with massive hemorrhage at delivery, spontaneous rupture remote from term has been increasingly reported in the literature.

In the present case, histopathology confirmed PAS involving the prior cesarean scar. The absence of antenatal care prevented early identification of abnormal placentation, which may have allowed for planned delivery in a tertiary care setting.

Extrauterine intact amniotic sac: a rare phenomenon

One of the most striking findings in this case was the presence of an intact amniotic sac containing the demised fetus within the peritoneal cavity. Comparable cases described are described in Table 1, where similarly reported preservation of the gestational sac following complete uterine rupture; however, extensive adhesion formation involving bowel structures and confirmed placenta accreta spectrum were not uniformly present. In contrast, the present case demonstrated dense agglutination of the intact extrauterine sac to the omentum, bowel loops, and anterior peritoneum, substantially increasing operative complexity and necessitating multidisciplinary intraoperative management. Furthermore, unlike some previously reported cases involving unscarred uteri, the present patient had two previous cesarean sections and histopathologically confirmed PAS involving the scar region, reinforcing the role of abnormal placentation and scar-related myometrial weakening in the pathogenesis of rupture.

**Table 1:** Summary of case reports of mid-gestational uterine rupture complicated by placenta accreta spectrum

Authors	Year	Gestational age	Uterine history	PAS type / placental pathology	Rupture details
Duggal BS, Khanna S. [12]	2006 (published 2006; indexed as 2011 in PMC)	~16 weeks (mid‑second trimester)	Previous three full‑term vaginal deliveries, first‑trimester MTP, and laparoscopic sterilization one year ago; likely silent left‑fundal scar from prior procedure	Placenta implanted in the left fundus over the scarred area; no histologic evidence of placenta increta or percreta	Spontaneous left fundal rupture in mid‑pregnancy with placenta protruding from the defect, active bleeding, ~1500 ml hemoperitoneum, and fetus still inside the uterine cavity; managed by subtotal hysterectomy
Dahiya, P. et al. [13]	2012	19 weeks	Two prior dilatations and curettages; hysteroscopic adhesiolysis (Asherman‑like adhesions); no prior cesarean section; IVF pregnancy after oocyte donation and embryo transfer	Placenta accreta (histology: chorionic villi reaching the myometrium with absent decidua basalis)	Spontaneous uterine rupture at 19 weeks with acute abdominal pain, haemoperitoneum (~3000 ml), fetal demise, left fundal defect with placental tissue firmly adherent to the myometrium; managed by subtotal hysterectomy for massive hemorrhage
Pal, S. et al. [14]	2014	21 weeks 3 days	G2P1L1; prior full‑term normal vaginal delivery; no prior D&C, cesarean, myomectomy, or other uterine surgery; no known prior uterine scars	Placenta percreta (chorionic villi invading myometrium up to serosa, confirmed on histopathology)	Spontaneous uterine rupture in the second trimester with whole‑abdomen pain and dysuria; intraoperatively, 2 L hemoperitoneum; fundus markedly thinned and cystic, with a 4×3 cm rent on the fundoanterior surface that extended and allowed fetal expulsion; placenta densely adherent and infiltrating myometrium and serosa; managed by emergency subtotal hysterectomy and massive transfusion
Seo, So. et al. [15]	2017	17 weeks (second trimester)	Multiparous woman (G2P1L1); prior normal vaginal delivery; no prior uterine surgery or cesarean reported (unscarred uterus)	Placenta percreta (histologically confirmed chorionic villi penetrating through full‑thickness myometrium to serosa)	Sudden severe abdominal pain and hypotension; laparotomy revealed ~2 L hemoperitoneum, a cornual fundal uterine defect with placental tissue fully penetrating the uterine wall, and intrauterine fetal death; managed by subtotal hysterectomy for massive hemorrhage
Katti, F.et al. [16]	2025	22 weeks	G3P2 with two prior cesarean deliveries, the latest 9 months before presentation (lower‑segment cesarean scar)	Placenta increta with central placenta previa; chorionic villi invading the myometrium and absent decidua basalis on histopathology	Sudden severe abdominal pain, hypotension, and shock at 22 weeks; ultrasound‑suspected rupture at prior cesarean site, ~2.5 L hemoperitoneum at laparotomy, non‑viable fetus; total hysterectomy performed due to massive hemorrhage and unseparable placenta from myometrium
Martel,K., Getto, L. [17]	2025	18 weeks	34‑year‑old G1 (no prior pregnancies reported in the abstract; no prior uterine surgery or cesarean explicitly mentioned)	Placenta percreta (histologically confirmed placenta accreta spectrum with full‑thickness myometrial invasion)	Acute abdominal pain, hemodynamic instability, and peritonitis at 18 weeks; bedside ultrasound showed significant free fluid with a live intrauterine fetus; intraoperative hemoperitoneum from uterine rupture secondary to placenta percreta; emergency hysterectomy required with massive transfusion protocol; patient survived and was discharged home
Mottaghi,M. et al. [18]	2026	20 weeks (mid‑gestation)	36‑year‑old gravida 9, para 5 with prior cesarean deliveries; rupture occurred along the previous cesarean scar	Placenta percreta (chorionic villi crossing full‑thickness myometrium into overlying serosa, with bladder invasion)	Spontaneous rupture of the anterior uterine wall at the site of prior cesarean scar, with extrusion of a nonviable fetus into the abdominal cavity and spontaneous disruption of the bladder due to placental invasion through the serosa into the bladder; managed by subtotal hysterectomy, left salpingo‑oophorectomy, and bladder repair; patient recovered but reported overactive‑bladder symptoms at 2‑year follow‑up
Drever, N. et al. [19]	2026	17 weeks	28‑year‑old multiparous woman with three prior cesarean deliveries; prior ultrasound at 17 weeks did not show sonographic features of PAS, but placenta previa was present	Placenta accreta spectrum (histologically confirmed abnormal placental adherence with invasion beyond decidua, though exact level of invasion—accreta/increta/percreta—is not fully specified in the citation metadata)	Massive haemorrhage during mid‑trimester medical termination with mifepristone and misoprostol; uncontrolled bleeding, haemodynamic collapse, intraoperative cardiac arrest, and emergency subtotal hysterectomy required; procedure complicated by dense vesicouterine adhesions and bladder injury; managed with massive transfusion and intensive care; patient survived
Mou, Y., Yang, H., Zhong, Q. [20]	2026	25–27 weeks (second‑trimester subset)	7‑case series: 4 ruptures in the second trimester; 3 involved prior cesarean scars, others had prior vaginal deliveries and varying uterine histories	Three cases complicated by placenta accreta spectrum (PAS), including placenta percreta and increta (2 in scarred uteri, 1 in a non‑scarred uterus)	Spontaneous complete uterine rupture in the second trimester, full‑thickness tear of endometrium‑myometrium‑serosa; rupture sites included lower uterine segment, fundus, and cornua; all PAS‑associated cases had massive hemorrhage and hemorrhagic shock, managed by subtotal or total hysterectomy or uterine repair; high perinatal mortality (6/7 fetal deaths), with only one live birth in late gestation

Only isolated case reports describe complete uterine rupture with preservation of membrane integrity [8,9]. In such cases, the fetus remains enclosed within the amniotic sac despite full-thickness myometrial disruption.

The proposed mechanism involves rapid rupture of the weakened uterine wall with expulsion of the gestational sac before membrane rupture occurs [8,9]. In some reported cases, the extrauterine sac has been found loosely positioned within the abdominal cavity. However, in more complex presentations, particularly when associated with PAS, dense adhesions to adjacent intra-abdominal structures have been documented [10]. In this case, the amniotic sac was densely agglutinated to loops of small bowel, the omentum, and the anterior peritoneum. The invasive placental pathology likely contributed to inflammatory reactions and adhesion formation. This finding significantly increased surgical complexity and required intraoperative collaboration with a general surgeon to safely divide bowel adhesions and prevent enteric injury.

Recent literature similarly suggests that PAS-associated uterine rupture outside labor remains a rare but increasingly recognized complication in women with multiple prior cesarean deliveries. Several recent reports have emphasized the importance of early antenatal diagnosis of PAS, as delayed recognition may contribute to catastrophic maternal morbidity, extensive hemorrhage, bowel involvement, and the need for hysterectomy.

Maternal and fetal outcomes

Maternal outcomes in spontaneous uterine rupture are primarily determined by the rapidity of diagnosis and intervention. Massive hemoperitoneum, hemorrhagic shock, disseminated intravascular coagulation, and need for hysterectomy are frequently reported. Perinatal mortality is particularly high when rupture occurs before viability or when diagnosis is delayed [1-3].

In second-trimester rupture associated with PAS, fetal demise is common due to acute placental separation and catastrophic uteroplacental insufficiency [4]. In the present case, ultrasound confirmed intrauterine fetal demise at presentation.

Prompt surgical intervention likely prevented further maternal deterioration. The patient required a subtotal hysterectomy and resection of the left adnexa due to uncontrolled hemorrhage. Despite the severity of the presentation, the postoperative course was uncomplicated, and the patient was discharged in stable condition after six days.

Surgical management and multidisciplinary approach

Emergency laparotomy remains the cornerstone of management in uterine rupture. In cases complicated by PAS and extensive myometrial destruction, hysterectomy is often the definitive and life-saving treatment [5,6]. Conservative uterine repair may be considered in selected cases where bleeding is controlled and placental invasion is limited; however, in the presence of significant hemorrhage and structural disruption, hysterectomy remains the standard of care.

The involvement of adjacent intra-abdominal structures in this case necessitated a multidisciplinary approach. The requirement for general surgical assistance highlights the complexity of managing PAS-associated rupture with bowel adhesions [6,11]. Coordination between obstetricians, anesthesiologists, blood bank services, and general surgeons is essential in such catastrophic presentations.

An important aspect of the present case relates to the clinical decision-making process during the initial presentation. At admission, the patient was hemodynamically stable, and ultrasound examination demonstrated intrauterine fetal demise without definitive sonographic evidence of uterine rupture. Consequently, initial management focused on induction following fetal demise while maintaining close inpatient monitoring. Retrospectively, occult rupture or progressive scar dehiscence may already have been present but was not clearly identifiable on initial imaging. The subsequent progression of persistent abdominal pain and recurrent vaginal bleeding prompted repeat ultrasonographic evaluation, which raised a strong suspicion for uterine rupture and led to immediate surgical intervention. This sequence highlights the diagnostic challenges associated with atypical presentations of uterine rupture and reinforces the importance of continuous reassessment in high-risk patients with previous cesarean sections.

Importance of antenatal surveillance

This case also emphasizes the critical importance of antenatal care in women with prior cesarean sections [7]. Early identification of PAS through targeted ultrasound and, when necessary, magnetic resonance imaging allows for risk stratification and planned delivery in specialized centers equipped to manage massive obstetric hemorrhage.

The absence of antenatal follow-up in this patient likely delayed recognition of abnormal placentation and prevented anticipatory management. As cesarean rates continue to rise globally, vigilance for PAS and related complications is increasingly important.

The surgical approach in this case was determined by the extensive structural disruption identified intraoperatively. Persistent hemorrhage, complete rupture at the previous cesarean scar site, significant destruction of the lower uterine segment, and dense adhesions involving surrounding bowel structures precluded conservative uterine repair. Therefore, subtotal hysterectomy represented the safest life-saving surgical option for hemorrhage control and prevention of further maternal morbidity. Intraoperative involvement of a general surgeon was also required because of the intimate bowel adhesions surrounding the intact extrauterine gestational sac, emphasizing the importance of multidisciplinary management in complex PAS-associated uterine rupture.

## Conclusions

Second-trimester uterine rupture remains a rare but potentially catastrophic obstetric emergency, particularly in women with multiple previous cesarean sections and undiagnosed placenta accreta spectrum (PAS). The present case is further distinguished by the exceptionally rare finding of an intact extrauterine amniotic sac densely adherent to intra-abdominal structures, significantly increasing operative complexity. Although the patient reported a preceding fall prior to symptom onset, operative and histopathological findings suggest that PAS involving the previous cesarean scar and underlying scar-related myometrial weakening were the principal predisposing factors for rupture, while the traumatic event may have acted as a precipitating trigger in an already structurally compromised uterus. This case highlights how the absence of antenatal surveillance may delay the recognition of abnormal placentation and increase the risk of severe maternal morbidity, including catastrophic hemorrhage and hysterectomy. It also demonstrates that uterine rupture may initially present with relatively nonspecific symptoms despite advanced intra-abdominal pathology, emphasizing the importance of repeated clinical and ultrasonographic reassessment in high-risk patients. Early diagnosis, rapid surgical intervention, availability of blood products, and multidisciplinary collaboration were essential for successful maternal stabilization in this complex presentation.
